# Democratizing science with the aid of parametric design and additive manufacturing: Design and fabrication of a versatile and low-cost optical instrument for scattering measurement

**DOI:** 10.1371/journal.pone.0187219

**Published:** 2017-11-07

**Authors:** Jose M. Nadal-Serrano, Adolfo Nadal-Serrano, Marisa Lopez-Vallejo

**Affiliations:** 1 Departamento de Ingeniería Electrónica, Universidad Politécnica de Madrid, Madrid, Spain; 2 Department of Software Engineering and Artificial Intelligence, School of Computer Engineering, Univ. Complutense de Madrid, Madrid, Spain; Beihang University, CHINA

## Abstract

This paper focuses on the application of rapid prototyping techniques using additive manufacturing in combination with parametric design to create low-cost, yet accurate and reliable instruments. The methodology followed makes it possible to make instruments with a degree of customization until now available only to a narrow audience, helping democratize science. The proposal discusses a holistic design-for-manufacturing approach that comprises advanced modeling techniques, open-source design strategies, and an optimization algorithm using free parametric software for both professional and educational purposes. The design and fabrication of an instrument for scattering measurement is used as a case of study to present the previous concepts.

## Introduction

Since 1970, 3D printing has experienced a great interest and a remarkable technological improvement. Fused Deposition Modeling (FDM) was developed by S. Scott Crump in 1988, which allowed to create three-dimensional objects by the successive addition of layers of fused material in a predefined sequence [1]. FDM is arguably the most known and extended 3D fabrication method worldwide today; in fact, it can be said that it has enabled further research on the basis of an unprecedented hardware democratization as a consequence of its relative simplicity and affordability combined with outstanding price-to-quality ratio.

The so-called Open Software Appropriate Technology (OSAT) [2] has the potential to harness the capacities of distributed peer review, software transparency, and the culture from the open source movement, the academic world, and the contextual development of Appropriate Technologies (ATs) all at once. In this particular ecosystem, users are free to use and modify the source from any shared AT and, via the internet, engage in a parallel world-wide peer review process to determine the best practices and solutions, which tend to self-organize and standardize.

Most AT-related, rapid prototyping-focused works refer to the use of desktop hardware and printers, but leave Parametric Design (PD) out of the equation. Parametric design allows for real-time design checking, unmatched adaptability and flexibility through easily controllable design drivers via rich user interfaces –such as McNeel’s Grasshopper [3] and other visual programming environments–.

Parametric Design software relates a series of highly customizable systems whereby the user is able to create interactive geometry-creation rationale and workflows with the aid of scripting and parameterization. Additionally, parametric-driven systems award high degree of customization, the affordability of fabrication, a very timely and predictable response when modifying designs, fabrication reliability, geometrical accuracy, and the scalability of parts and models. Moreover, users are –as a result of the availability of 3D printing devices and appliances– able to produce models in a delocalized fashion, creating alternative fabrication networks to current, regular commercial manufacturing streams exposing a much wider audience to scientific research until now unreachable due to economic reasons.

The present paper proposes a fully-parametric approach to the design-for-fabrication of an open-source model using Additive Manufacturing (AM) for rapid prototyping while conveying the design and assembly of a totally expandable, easy-to-use, and affordable optical measurement instrument used as a case study.

The approach hereby discussed presents a low-cost instrumental setup created using additive manufacturing and off-the-shelf –though adaptable– components, electronics, etc. The design stems from the self-imposed need to host purely FDM-printable parts together with other affordable and standardized parts, which must meet the condition to become part of a screw-less assembly. Furthermore, the presented approach assumes “open-sourceness” for both its hardware-fabrication technology and its parametric-oriented design through an openly accessible knowledge platform: all the designs and the source code of the parametric system are made available online or upon request.

Some of the most outstanding features of the whole Design-to-Production (D2P) workflow presented in this paper and its associated algorithm are:

- The modularity of the system, which allows for quick response to iterative design changes and to reuse parts for future instruments and setups, allowing their easy variation in shape, size, and material conditions.
- The development of an adaptable and extendable design-for-fabrication workflow, emphasizing the simplicity of the experimental setup without sacrificing accuracy.
- The evaluation and inclusion of low-cost parts and materials in order to maximize performance while further lowering fabrication costs and times.
- The employment of advanced 3D modeling and additive manufacturing techniques to create custom, specific parts needed, matching the evolving requirements of the setup itself.
- The invention of an easy-to-use system to guarantee maximized user response and maximized system accuracy, both in terms of design and fabrication.

The idea of customizable instruments [4] or –more precisely–, the use of additive manufacturing to create custom optical gear has been explored before, with authors having contributed open source libraries [5]. Nevertheless, this paper introduces parametric design in the design flow as a novelty, demonstrating the viability of the whole process through the design and fabrication of a functional instrument.

The paper presents the design and fabrication of a scattering measurement instrument, which will serve as a case of study and a practical means to present the aforementioned concepts. The complexity of the instrument will be shown in the next sections along with its optical and electronics subsystems.

The paper is divided into several sections. The first one gives an overview of the design and concept of the instrument pursued. Then, the setup and conception of the design from a parametric D2P perspective is presented, emphasizing the description of the parametric algorithm developed. Some remarks on the fabrication are done next, where the relative strengths and weaknesses of a proof-of-concept setup are analyzed. Last, some conclusions on the overall process are drawn.

## Concept and design overview

The case of study, an instrument to measure scattering is presented. Scattering has been one of the most extensively studied phenomena in Physics in the last two centuries. Numerous theories have been developed to explain scattering under certain circumstances, giving birth to Mie, Rayleigh, Raman and other types of scattering. There is still extensive research on the topic going on and so there is a need for instrumentation to validate new computational models.

The design and realization of the instrumentation and the related experiments must give and take strategy, in order not to affect –or affect the least possible– the phenomena under study and simultaneously obtain reliable data. A trade-off has to be made to ensure accuracy, correctness and reproducibility while keeping costs and timing reasonable.

For example, instruments to measure macroscopic optical coefficients on highly turbid media have been published in recent times [6]. Others, such as [7] have focused on low-level turbidity in water or have used other techniques like multisprectral analysis [8] or dynamic light scattering (DLS) techniques [9] to measure nanoparticles in water.

An optical incident radiation in a form of a very narrow beam will travel from the emitter –located at one end of the instrument– to the receiver at the other end. In its trip, the propagating radiation will get through a medium (a particle-filled atmosphere) where it will be scattered. The resulting measurement will be made at the receiver end with an appropriate sensor.

**Fig 1:**
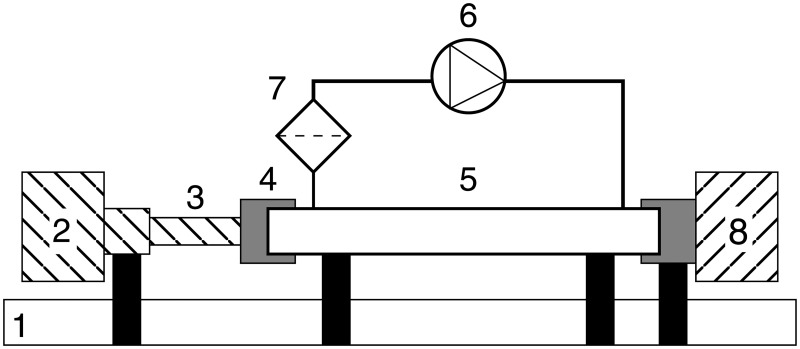
System overview. 1. Optical bench and supports, 2. Emitter and driver electronics, 3. Optical hardware, 4. Pipe ending and joint, 5. Confined space for the medium, 6. Medium injector, 7. Air filter, 8. Sensor and data acquisition.

**Fig 2:**
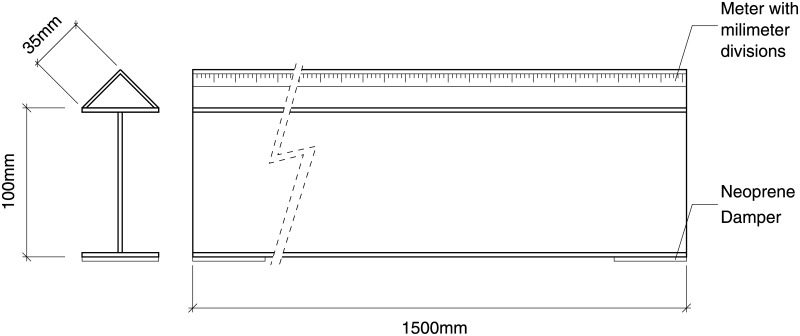
Custom optical bench.

**Fig 3:**
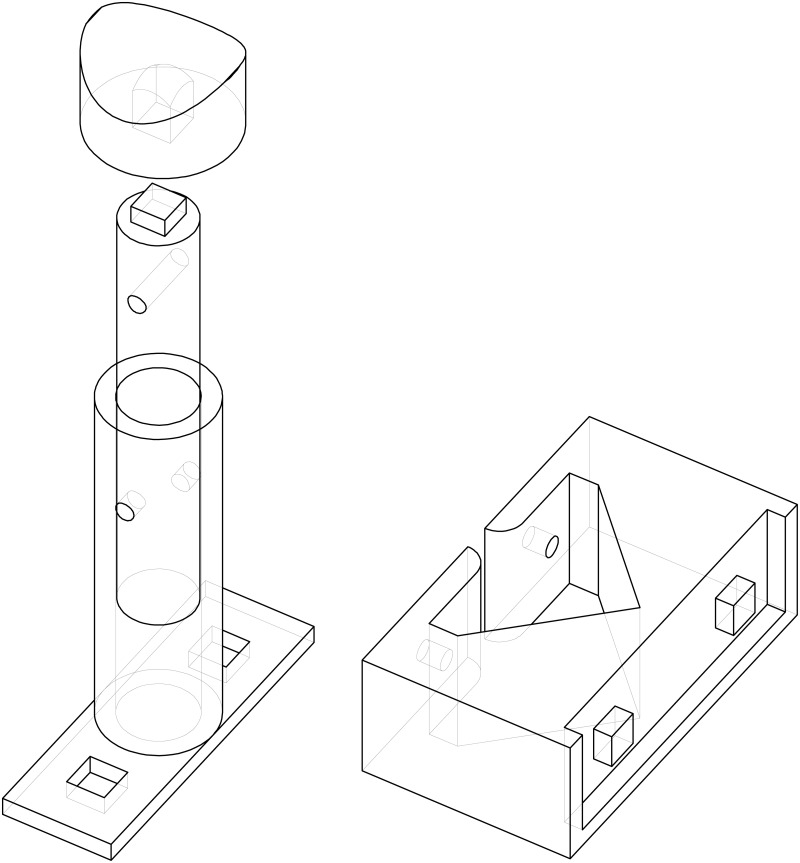
Support parts. Base (right); Base cylinder, inner cylinder and holder (left). The inner cylinder has been put into the base cylinder for illustration purposes.

**Fig 4:**
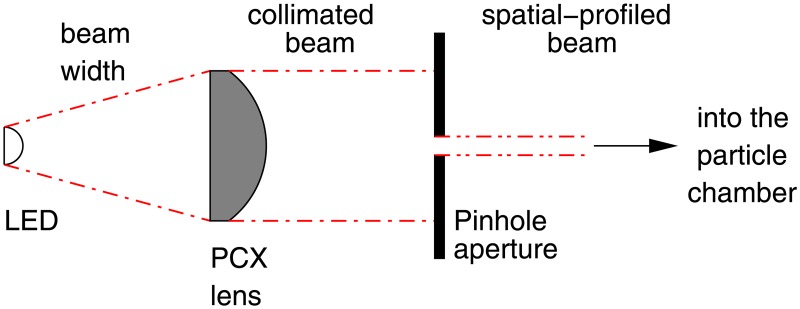
Collimation and spatial profiling of the optical radiation. The schematic drawing shows the PCX lens and the pinhole aperture.

**Fig 5:**
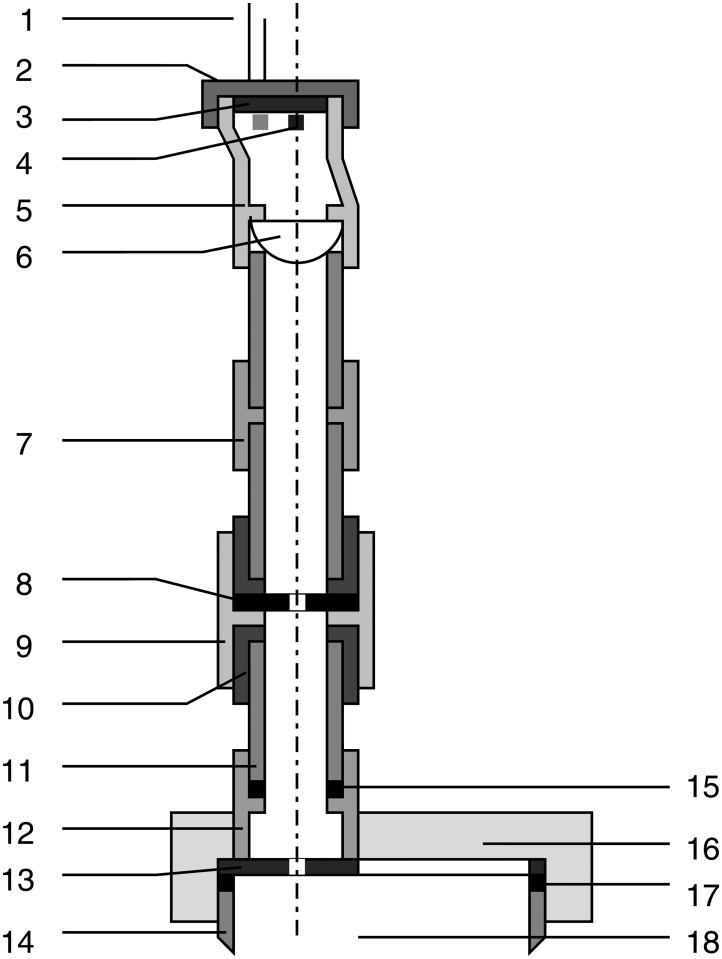
Optical radiation subsystem (half section). 1. LED connectors; 2. LED PCB holder; 3. LED PCB; 4. Active LED alligned (other LEDs marked in grey); 5. Eccentric part; 6. 20mm PCX lens; 7. 20mm PVC socket; 8. 10*μ*m pinhole; 9. 25mm PVC socket; 10. 25/20mm PVC reducer; 11. 20mm PVC pipe; 12. 20mm PVC socket; 13. Microfilter and chamber interface; 14. 50mm PVC pipe; 15. EVA sponge seal; 16. 50mm cover for microfilter; 17. EVA sponge seal; 18. Particle chamber.

**Fig 6:**
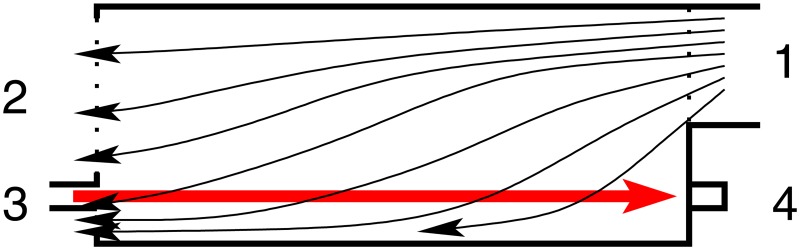
Air and light flux directions inside the particle chamber. Air flux shown with thinner arrows, light flux with one thick arrow: 1. Air inlet; 2. Outlet (with microfilter); 3. Light inlet; 4. Light sensor.

**Fig 7:**
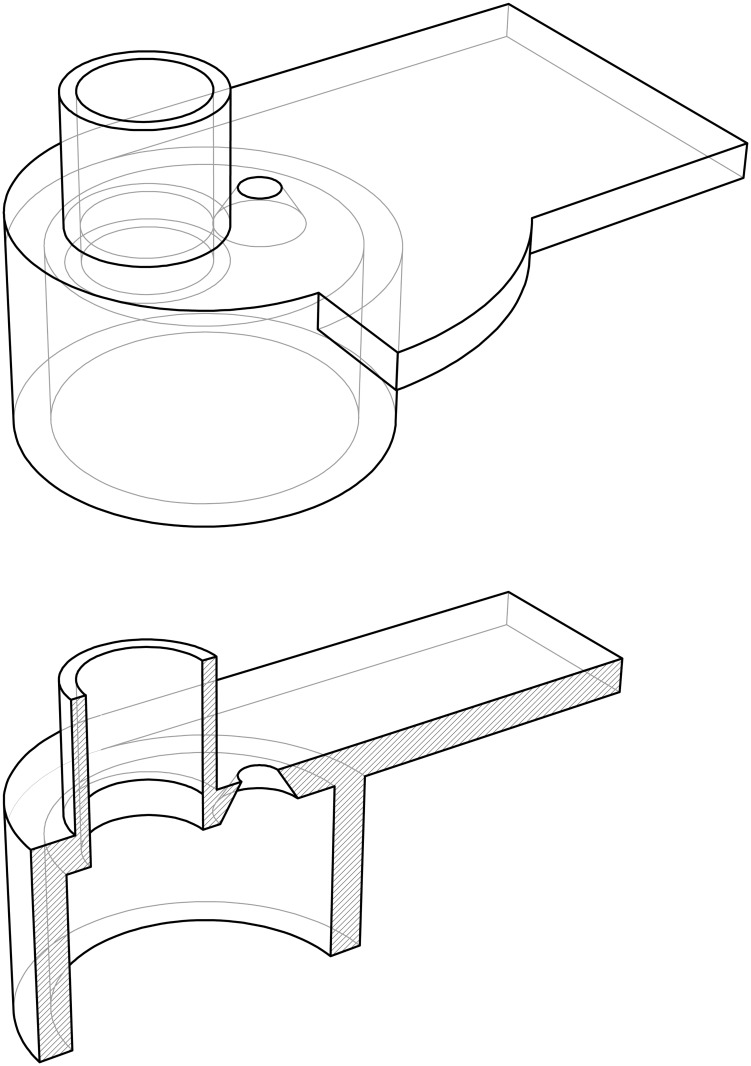
Pipe ending with camera holder. The opening for the camera corresponds to the conic aperture.

The proposed instrument consists in a series of elements aligned so that the optical radiation goes from the emitter to the sensor. A visual introduction to the design and aim of the instrument itself is available in S1 Video. A simple scheme of the necessary elements is depicted in Fig 1:

1. **Optical bench and supports**. An optical bench was designed and fabricated ad-hoc for the experiment in a search for a custom and tight budget solution. A steel-made European standard IPN100 beam was covered with a 35mm stainless steel right angle structure on top and cut to the task. A meter with milimeter divisions was added as well to be able to record and reproduce the position of the holders for the experiments. The design is shown in Fig 2.
  A number of supports were designed to match the optical bench. They were designed in blocks following a “design expandability” philosophy. The supports consist in a set of four parts (base, base cylinder, inner cylinder and holders of several types), designed so that few designs could be combined to build many different setups as seen in Fig 3.
2. **Emitter and driver electronics**. A narrow light beam is needed for the experiments. Hence, high-power LEDs were chosen for the ilumination of the scattering medium: they emit narrowband light for a wide range of wavelengths at affordable prices and do not suffer from speckle noise.
  The design of a special-purpose eccentric part in combination with a custom PCB made it possible to conduct up to three different experiments (each one corresponding to a different optical source) with the same scattering medium in a very short time. This enabled quality comparison among results obtained for different optical sources when illuminating the same scattering medium. Custom current drivers for the high-power LEDs were also designed. The schematic is depicted in S1 Fig.
3. **Optical hardware**. Its purpose is to conform the radiadion pattern to obtain a light beam as narrow as possible. This process involves two steps: collimating the beam and spatially profiling it (see Fig 4). A pinhole aperture of 10*μ*m in diameter was used for the experiment, a size comparable to a pixel in many CMOS cameras.
  The optics and the electronics were assembled together into a compact holder to be attached to the particle chamber, resulting in a system capable of generating a micron range-wide light beam from a high power LED.
  Optical and electronic subsystems were put together using high pressure PVC pipes and parts, as they are widely available, cheap and provide a perfect fit for the pinhole aperture and the lens. The scheme is shown in Fig 5. The light emanates from the LED positioned in 4 and goes all the way down to 18 through the lens and the pinhole.
  Finally, the electro-optical subsystem must be coupled to the particle chamber. A system to prevent the particles in the chamber from leaving while allowing the optical radiation in was designed, using custom covers.
4. **Confined space for the medium**. A 50mm diameter PVC high pressure pipe was decided to be a good trade-off between internal volume, size and versatility at a reduced cost. This way the scattering path can be easily varied using pipes with different lengths. In addition, its round nature would prevent particles from accumulating at the corners.
  The pipe serving as the particle chamber needed special endings to allow light and particles (scatterers) in. In addition, particles must not leave the chamber once injected and light must traverse the chamber only affected by scattering.
5. **Medium injector**. Particles are injected into the chamber traveling inside microscopic droplets of similar size. The use of a convenient carrier ensures rapid evaporation, leaving only the desired particles. A medical nebulizer (PARI LC) was used to the task.
6. **Air filter to prevent smoke particles from leaving the medium chamber**. The particles injected in the chamber must be confined to avoid possible leaks. Therefore, we designed a system which granted a constant air flux while these particles are injected. A 47mm MILLIPORE 0.22*μ*m membrane filter (GSWP 047 00) was installed to avoid particles from escaping the chamber.
  Light traveled in the opposite direction to the air flux to ease manufacturing, measurements, and air flux itself (see Fig 6).
7. **Sensor and data acquisition**. Data acquisition is an essential part of any experiment. For scattering measurements, a sensitive and fast profile sensor or digital camera, with as low acquisition times as possible and reduced sensor area is pursued.
  The search for alternative options combining high frame rate with a suitable form factor at a reasonable accuracy and cost resulted in a search for digital cameras integrated in mobile phones. Several models were tested and finally an iPhone 6 working in slow motion mode at 240fps was chosen for the task.
  Fig 7 shows the pipe ending including the camera holder.
8. **Data gathering and processing**. The data obtained with this instrument (mainly still images, although videos can be also obtained) can undergo further postprocessing to enhance their analysis and interexperiment comparison.

## Parametric D2P: Setup and conception

As explained above, the main elements of the experiment consist of an optical bench, a series of electronics, and a physical device distributed in a series of blocks. Whereas the design of the electronics and the optical hardware remained constant for the duration of the project, the optical bench and supports, along with pipe terminations and washers involved constant modifications and testing, sometimes even requiring daily updates in a full customization framework.

Traditional fabrication techniques –such as mechanization, molding, plastic injection, and others– were too costly and slow for our needs. Consequently, a parametric system was adopted whereby time, material, and cost derived directly from an automated design logic materialized through a geometry-generation algorithm.

The **premises** of the parametric system adopted are the following:

- It must be able to create an automated geometry following a series of design guidelines implemented by a parameter-driven design.
- It must provide valuable, reliable, design-time information (prior to exporting the model to any slicing or similar software) about fabrication setup, times, and cost with little or no input from the user.
- It must be adaptable and conclude in a printable setup according to the limitations of most desktop printers, considering their maximum printing capacity and size.
- It must be easy to use and configure.

Fabrication procedures have been kept in mind in all design phases. On account of the parametric design it was possible to automatically adjust real tolerances of 3D printed parts taking the actual extrusion hardware and process into account. Parts benefit not only from a higher quality finish and precision, but also from direct assembly without the need for external or auxiliary means: parts were conceived to fit tightly, precisely, and exactly through pressure.

From all the parts designed, supports and pipe gear are indubitably the most prone to modifications, amendments, and adjustments. As such, a component-specific algorithm which controlled the geometry, shape, and infill was developed for the following parts: support base, base cylinder, inner cylinder, and the pipe holder.

The algorithm allows the user to load or adopt any profile shape, which is then extruded a length according to another user-defined parameter.

### Design algorithm

The algorithm works in a sequential, multi-phase manner. A general view of the main algorithm blocks is available in S2 Fig. As the geometry of certain parts depends on others, the calculation is realized according to the falling dominoes logic: first, the base is calculated according to the user-defined profile; second, special parts designed only for calibration purposes were fabricated in an iterative process aiming for perfect fitting and maximizing accuracy while minimizing material, time and energy cost. This simple idea yielded considerable savings.

Different parts yield different results in terms of material optimization, which is achieved at mainly two levels: first, parts are built without producing waste –only the exact amount of material required for fabrication is used–. Second, the infill was adjusted to comply with the function of each part, thus saving from 20% to 80% depending on the parts. For instance, the main cylindrical base saves up to 40% of material, whereas secondary parts related to electronics save 80%. Furthermore, it is possible to implement high-level structural analysis on printable parts in order to optimize their structural behaviour and to characterize their infill patterns [10]. Therefore, application-dependent but significant cost reductions can be achieved using the method presented here, coming not only from material savings (in the 20–80% range when compared to an all-solid part), but also from timing (the process is controled by the user from design to fabrication). Besides, the user has the possibility of creating full custom designs made combining commercial with custom manufactured parts.

Grasshopper allows to reference any geometry from a Rhinoceros 3D model. This feature is taken advantage of in order to implement the flexibility required for the design, allowing the user to either pick a pre-existing base profile or to manually draw a customized one. Grasshopper calculates the whole algorithm every time a single parameter value is modified. Moreover, a customized user interface is provided in order to ease the use of the program, preventing the user from going through unnecessarily complex software learning processes. The process described here could be easily translated into different software packages for the best user’s convenience. S2 Video shows the interactive process.

#### Common algorithm parameters

In addition to design parameters, the user is prompted to enter some common and 3D printing-related parameters, such as:

- **Extruder diameter**: normally 0.4mm. It is used to calculate the part’s tolerance when they interlock. This tolerance must be no less than the extruder radius plus 0.05mm and no more than the radius plus 0.075mm. For parts sharing a single edge, the former tolerance may be reduced to the radius plus 0.025mm or even less.
- **3D printing variables**: pre-printing information is also made available by the algorithm as a means to control the fabrication in real design time. The algorithm displays approximated printing times through an easy calculation of the toolpath length based upon these parameters:
  - Number of shells, ranging between 1 and 3 for most parts.
  - Layer height (measured in mm), normally between 0.1 and 0.2.
  - Printing speed (in mm/s): usually 50. Please note that no traveling speed is provided, which affect the final results. These must be considered demonstrative. Nonetheless, the simulation run by the algorithm is precise enough and up to 20 times faster than a real calculation carried out by the well-known Slic3r or Cura Engine software packages.
  - Solid proportion of infill in solid parts, which very much depends on the use of the part. Although interesting research has taken place for material optimization purposes [10], the infill here would depend on the final slicing software employed for fabrication.
  - No support material is taken into account, as none is needed for the fabrication of the parts.

#### Per part detailed description and semi-automated design

In the following paragraphs, a detailed description is done of the semi-automated design and practical hints for the parts in Fig 3 and some other auxiliary ones.

#### The base

Different shapes result in different solid extrusions, yielding a different number of faces per solid shape. The user may draw a single profile curve anywhere in the drawing and then reference it in the Grasshopper definition. The profile will then be automatically re-oriented to match best the automated geometry, and extruded as desired. This may result in virtually limitless designs depending on the ultimate intent of the project.

As the support cylinder is intended to be oriented vertically, the user must manually pick the face on top of which he wants the cylinder base to be placed. Then, the algorithm calculates automatically the center of the selected face, and creates two boxes along its longitudinal axe, given their size and distance between them –user inputs–. These two boxes are increased in size by the algorithm according to the given tolerance in order to create the female connector in the cylinder’s part. Furthermore, a solid is extracted from top of the profile-based extrusion as to provide an overlap between the base and the cylindrical base given a certain margin. The male connectors are then moved to place automatically.

The same general design principles and physical concepts used with other fabrication technologies apply here. During the course of the tests, the **base** was found to work best using a width within a range of 50 to 100 mm for this particular design, as it allows for maximized part stability while enabling the parts to absorb geometrical imprecisions deriving from the bench itself. For other setups it could be necessary to scale the parts or use different proportions.

#### The base cylinder

The tube part basically consists of two components: its solid, rectangular base (platform), and the tube itself (hosting the support). The rectangular base derives directly from the geometry described above, whereas the tube is drawn upon the center plane of the upper-most face of the box-shaped platform. The platform serves as interface between the cylindrical support and the base.

The base cylinder must be easily attachable and detachable from the base mount. For that purpose, two box-like female connections are provided that fit tightly into the base’s male connection. The two connectors are subtracted from the platform solid through a simple Boolean operation. Based upon the male box connectors, another auxiliary two are created. These are scaled up to match the tolerance, and then used for the subtraction so as to obtain the female connectors. The tolerance is considered to be half the extrusion diameter plus 0.05mm for the E3D V6 extruder used by the printer –other hardware configurations may need to vary this number, although it is also possible to easily access this figure in the algorithm–.

The tube is defined through an outer and an inner radii. Grasshopper offers a variety of implementation options for cylindrical geometry, the most suitable of which takes a plane and radius as input parameters. While the radii are easily accessible by the user through a wide range of input components—see Grasshopper’s panels, sliders, or number parameters–, the base plane must be calculated automatically by the algorithm. The base plane for the cylinder coincides with the host face’s, situated exactly at its center of mass. This center is pre-calculated in a “mass” component and used as input parameter. Finally, the height of the cylinder can also be easily determined by the user through conventional input methods. Moreover, the user can establish the number and radius of a series of holes created along the cylinder with the purpose of providing a higher degree of flexibility or to ensure increased fixation strength through pins or other means. Their orientation is pre-established in order to facilitate their use once the parts are in place, thus placed perpendicularly to the longitudinal axe of the particle chamber. This “dovetailing” design proved to work in a reliable, flexible, and easily interchangeable manner as desired.

The base uses 40% of infill material and 3 shells in order to optimize its structural behavior and production times. Shells are required in order to protect the integrity of the base from the friction with the bench and to allow the part to absorb welding cord inaccuracies of the bench beam while keeping enough grip. Finally, an asymmetrical cylinder fixture is designed for unforeseen cases –note that base top displays only tabs along three edges; the tab width is also adjustable by design–.

The parameter values found to work best here were:

- Base height: 5–7.5mm.
- Outer and inner radii, respectively: 20 and 16 mm, which ensures maximized material inertia and strength minimizing infill material.
- Number and radii of pinholes: optional. Despite the fact that they are not required for the proper use of the system, they provide extended fixation capabilities and an air escape when it becomes necessary to extract the inner cylinder. A vacuum effect between the base cylinder and the inner support was found to take place due to the tightly adjusted system tolerance which in certain conditions made it difficult to manually remove the inner cylinder.
- The radius of the inner cylinder is derived automatically from the base’s hollow tube, also taking a total of 0.25mm of tolerance into account along its perimeter. Likewise, the cylinder’s height is adjustable through the algorithm’s user interface, granting the best results and control of the pipe’s horizontality with 200mm. It was also attested that, for the system to become stable for long testing sessions, at least 75mm of overlap were necessary for the cylinders to stay in place.
- Two different configurations were explored for the pipe base: either longitudinal or crossed. The longitudinal version must be purposely fixed on top of the inner cylinder, while the crossed configuration is symmetrical, allowing for a less careful positioning. Nonetheless, the first configuration is preferred, as it delivers greater stability. The user may define the radius of the pipe, 25mm in the present case. Since FDM was the preferred technology for building the parts, the female connector provided in this base has a “dome-like” finish in order to avoid the need for support material during the printing process. Again, the size of this connector is automatically calculated making use of the above logic and tolerances, which are calibrated and adjusted automatically in the geometry-generation algorithm.

#### The inner cylinder

The friction between the tube and the inner support was not only cause for the design of an air exhaust system, but also a phenomena that was further studied and taken advantage of adjust and fine-tune the design tolerance between interlocking parts of various natures. In this sense, the tolerance was reduced to the extruder radius plus 0.025mm, which worked perfectly for the round-shaped supports –lesser tolerance is admissible in parts having no corners or “kinky” shapes–. The top cap of the cylinder hosts a box acting as a male connector for the final pipe base.

#### The pipe base

The algorithm obeys a domino scheme. Consequently, most design constraints have been defined beforehand in preceding stages. The base responds only to two variables: its height and type. The height can be entered directly by the user, while the type is a binary value defining whether its shape will only accommodate a single or two crossing pipes. This is achieved by creating a set of two perpendicular pipes, and obtaining all possible subsets of the intersecting geometry. If only the longitudinal section is used, then type equals 1, otherwise type equals 2. The type controls the visibility of the final part.

#### The pipe endings

Pipe endings are very important parts of the design, as they are responsible for making the medium chamber “particle-tight” but at the same time allow particle injection and the propagation of light through the chamber. The algorithm takes the PVC pipe radius, along with the nebulizer outlet and the camera opening radii to generate one of the endings. The opposite one is created using the radius of the optical subsystem gear and the corresponding tolerances.

#### The washers and microfilter holder

The washers and microfilter holder inherit their properties directly from the pipe endings generated, as they take their inner diameter and the fabrication tolerance as input parameters. The small aperture needed for the ray of light to enter the medium chamber is made as small as the fabrication technology allows (in this particular case, 0.15mm in diameter).

## Fabrication of the parts

With the parts already designed, it is possible to add some additional optimization to the fabrication process. For instance, it is important to consider the properties and placement of supports where needed (the need for supports has been reduced to the minimum, and are only present in the pipe endings).

The printers used for fabrication were GCode based. Software such as CuraEngine [11] and Repetier [12] were used for this task. Parameters such as printer type and nozzle diameter (tolerance adjustments) have to be configured as well.

Different materials were tested during the design and production of prototype samples. Among other materials, thermoplastics such as ABS, PLA, and Carbon-Reinforced PLA were chosen to perform the tests. On the one hand, ABS displayed an undesired effect called “warping”, which affected the final geometry negatively when printing big parts. This effect is consequence of the significant material retraction and might be amplified if the printing atmosphere is not controlled properly. In other words, it was discarded due to technical constraints, such as the need for a hot bed and enclosed printing chamber. Since the design was thought to be open and accessible to any user, ABS-capable printers proved to be excessively complex.

On the other hand, PLA’s printing behaviour was reliable and easy to set up, displaying only minor or no printing defects. Additionally, PLA required neither a hot bed nor an enclosed chamber, which makes the technology more accessible to end users, which can buy PLA from a variety of firms online.

Moreover, Carbon-Reinforced PLA was tested for material optimization purposes, as the material was expected to have a better structural behaviour than its more basic counterpart. Nonetheless, parts did not perform significantly better, as the printing setup affects the structural soundness of the part even more than the material itself [13].

Finally, 1.75mm PLA was the material chosen, and the printer a Makerbot II [14]. A layer height of 0.15mm was used for maximum quality. CuraEngine configurations are available as source files in S1 File.

In addition to the placement of the individual parts for support minimization, it is important to pay attention to the layout in the printing bed when combining several parts to be printed at once. Not only the 2D placement, but the 3D shape of the parts must be taken into account. The aim is to minimize the number and length of extruder travels: a slight improvement in placement means significant printing time reductions. At this point there is no specific software for automatic placement of parts for 3D printing. This is left as future work.

## Proof-of-concept setup

This section shows the results obtained in the light scattering experiment conducted as a proof-of-concept. The sensor used was the iSight camera of an iPhone 6 and the particle chamber was filled with single-sized particles forming a uniform and low density atmosphere. S3 and S4 Figs show the exploded view of the instrument designed and a photograph of the actual setup.

**Fig 8:**
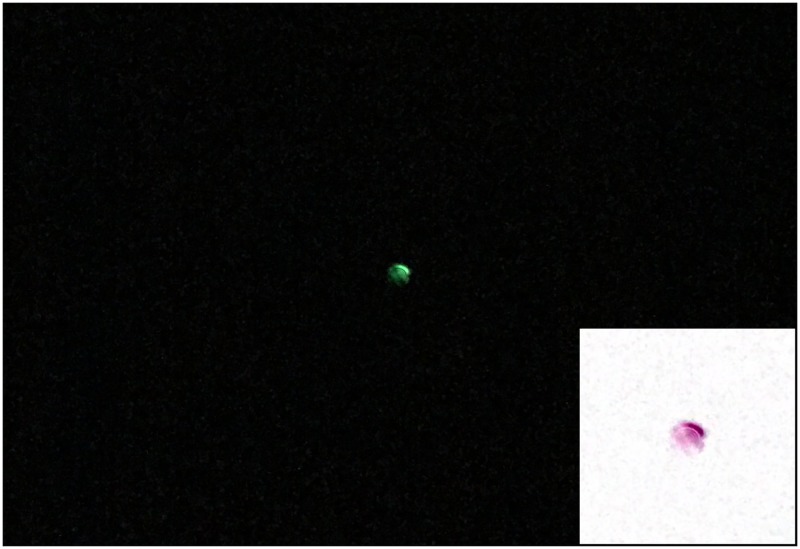
Example photograph taken during the experiments. Experimental results for 200000*μ*m mean free path, 1*μ*m particle size and 543nm illuminating wavelength. The center of the images has been magnified and colors inverted in the bottom right corner. Unprocessed image from the sensor.

Fig 8 shows the image obtained at the camera for a light atmosphere build using 1*μ*m spherical particles, in such a number that the resulting mean free path between particles is 200000*μ*m (that yields an average of two interactions per photon trip for a 400000*μ*m or 40cm light path). The radiating source is a LED with a peak wavelength of 543nm. In these conditions and given the sensitivity of the sensor used, the only pixels that receive enough radiation to show up in the picture are those corresponding to the direct transmission of light.

It is possible to obtain qualitative as well as quantitative values, using higher sensitivity calibrated profile sensors or sensors offering the raw data acquired. Also, more powerful light sources could be used.

## Conclusions

In the previous sections, a method to design and fabricate a system to measure scattering involving parametric design has been described. Key trade-off factors such as cost, feasability or design for fabrication have been present from the preliminary ideas to the final realization of the system. In the end, a simple but effective scattering measurement instrument has been created.

Several conclusions can be extracted from the whole process:

- Careful identification of the key design variables and other conditions that may affect the design at later stages is crucial to avoid having to redesign the prototypes. The “design for fabrication” philosophy is interesting when time frames and costs are tight.
- The combination of off-the-shelf with ad-hoc parts can have interesting benefits as well. In our case, the use of PVC standard pipes –cheap and easy to adapt to our necesities– with full custom 3D printed parts, provides big synergies without loss of accuracy if the basic concepts of the physical requirements of the experiment are clear.
- The use of 3D printing as a technique, and the possibility to apply the principles of parametric design to prototyping have shown clear advantages: the tolerances can be minimized using special-crafted probes. The results obtained can then be fed into the parametric model without having to redesign the whole 3D model saving time and resources.
- At this stage, the accuracy achieved by the instrument is not limited by the design and manufacturing process, but by data acquisition procedures and hardware capabilities. Also light beam generation and injection has strong implication in the type and density of the scattering media that can be studied. The use of better systems, such as fast and sensitive profile sensors or higher power light sources with ad-hoc designed lenses could improve the quantitative quality of the results obtained.

A simple system as it is, the proposed system provides with a cost-effective solution that can be applied in a wide range of applications. Moreover, the design methodology followed and its modularity has shown promising sinergies for future developments in the OSAT field.

## Supporting information

S1 Fig Custom combined arduino-controllable current driver for the high-power LEDs. The use of high power LEDs needs powerful current drivers controllable by an Arduino^®^UNO for higher customization. The circuits made use of an LM317, a well-known voltage regulator able to drive currents up to 1.5A. The LM317 fixes 1.25V between *V*_*o*_ and *V*_*adj*_. This made it easy to drive a LED, as *I*_*adj*_ is almost negligible.
(EPS)

S2 Fig Block diagram of the algorithm. A blockwise, general view of the algorithm is provided showing input, processing and output stages along with their associated settings and parameters.
(PDF)

S3 Fig Expanded view of the instrument designed for the scattering measurements. The numbers therein correspond to the following parts: 1. Optical bench. 2. Base holder. 3. Intermediate holder. 4. Inner cylinder. 5. Linear holder for 50mm pipe. 6. Sensor (digital camera). 7. Pipe ending with camera holder and nebulizer inlet. 8. 50mm diameter EVA washer (seal). 9. 400mm long, 50mm diameter high pressure PVC pipe. 10. 50mm diameter EVA washer (seal). 11. Microfilter holder and washer [1/2]. 12. Microfilter: 47mm MILLIPORE 0.22*μ*m membrane filter (GSWP 047 00). 13. Microfilter holder and washer [2/2]. 14. 50mm diameter EVA washer (seal). 15. Pipe ending with optical inlet. 16. 20mm diameter EVA washer (seal). 17. 20mm long, 20mm diameter high pressure PVC pipe. 18. 25/20mm high pressure PVC pipe reduction. 19. 25mm high pressure PVC pipe socket. 20. 10*μ*m pinhole, mounted (25mm diameter). 21. 25/20mm high pressure PVC pipe reduction. 22. 40mm long, 20mm diameter high pressure PVC pipe. 23. 20mm high pressure PVC pipe socket. 24. 40mm long, 20mm diameter high pressure PVC pipe. 25. 20mm diameter PCX lens. 26. Eccentric part for experiment optimization. 27. LED PCB with connectors. 28. LED PCB holder and cover.
(EPS)

S4 Fig Photograph of the actual experimental setup. (TIFF)

S1 File Source code. An archive with all the source files is attached. Please recall that they are available under GPLv2 or later.
(ZIP)

S1 Video Instrument overview. An overview of the aims and research framework of the instrument proposed is explained in detail in this video.
(M4V)

S2 Video Propagating changes in 3D models using parametric design. The video shows how changes in the design options are taken to the 3D models.
(M4V)
