# Supplementary figures and images for: Democratizing science with the aid of parametric design and additive manufacturing: Design and fabrication of a versatile and low-cost optical instrument for scattering measurement
Source: PLoS One. 2017 Nov 7;12(11):e0187219. doi: 10.1371/journal.pone.0187219 (PMC5675403; doi:10.1371/journal.pone.0187219)

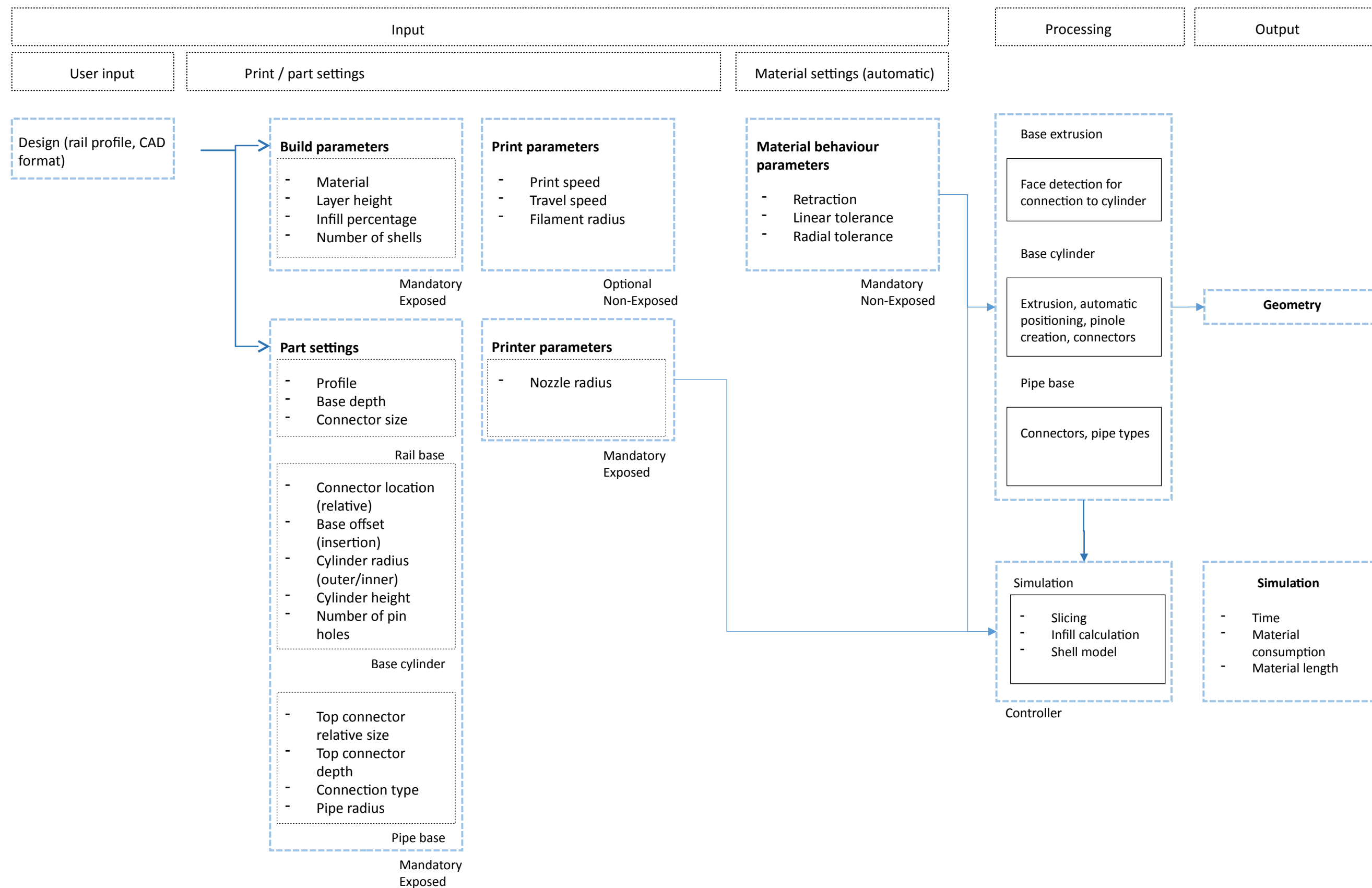

Supplement: S2 Fig — A blockwise, general view of the algorithm is provided showing input, processing and output stages along with their associated settings and parameters. (PDF) [file pone.0187219.s002.pdf]

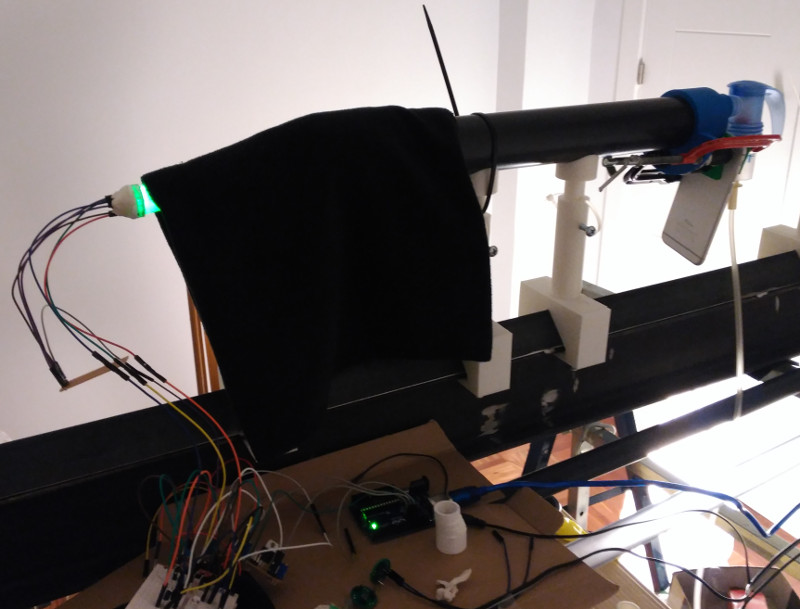

Supplement: S4 Fig — (TIFF) [file pone.0187219.s004.tiff]

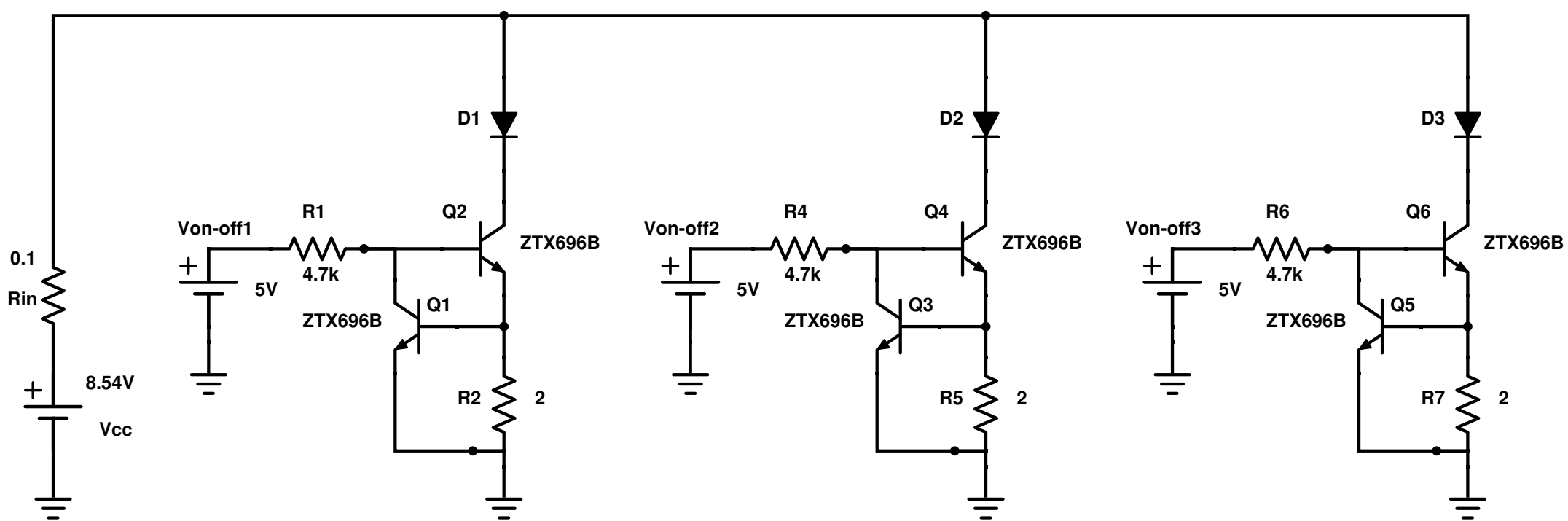

Supplement: S1 File — An archive with all the source files is attached. Please recall that they are available under GPLv2 or later. (ZIP) [file pone.0187219.s005.zip › S1-File/03-LED-drivers/LED-driver-common-plus.pdf]

# LED driver Simulation

PartSim Simulation Client

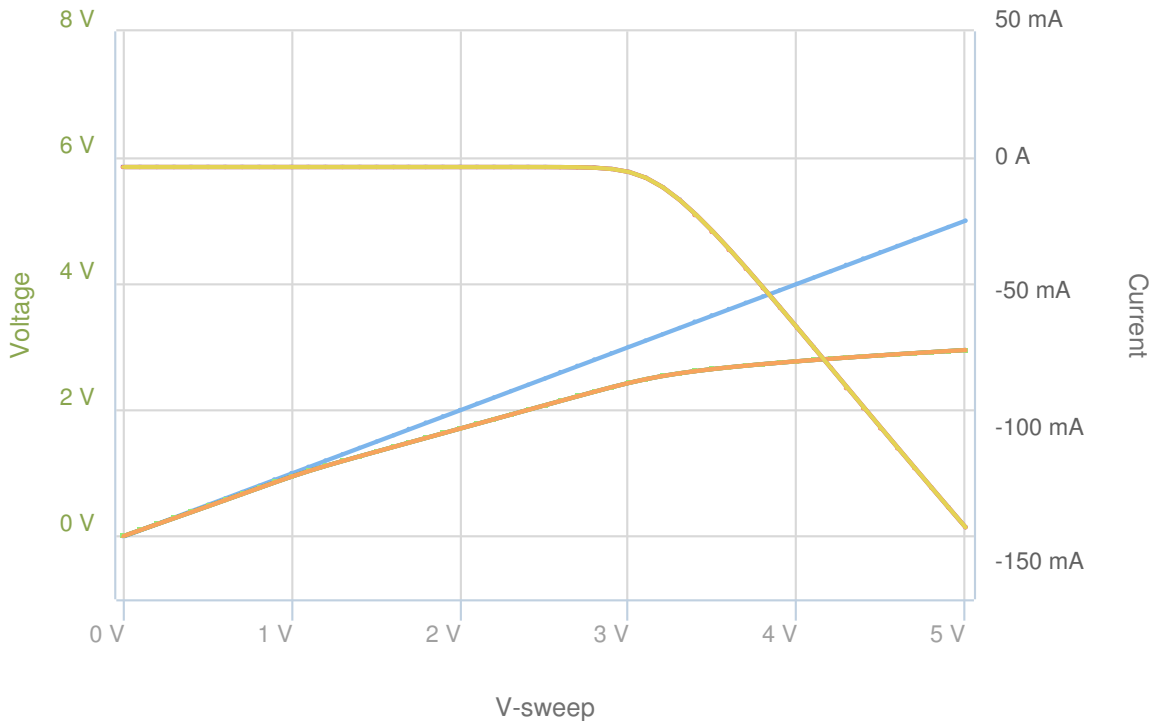

Supplement: S1 File — An archive with all the source files is attached. Please recall that they are available under GPLv2 or later. (ZIP) [file pone.0187219.s005.zip › S1-File/03-LED-drivers/LED driver Simulation DC transfer characteristic.pdf]

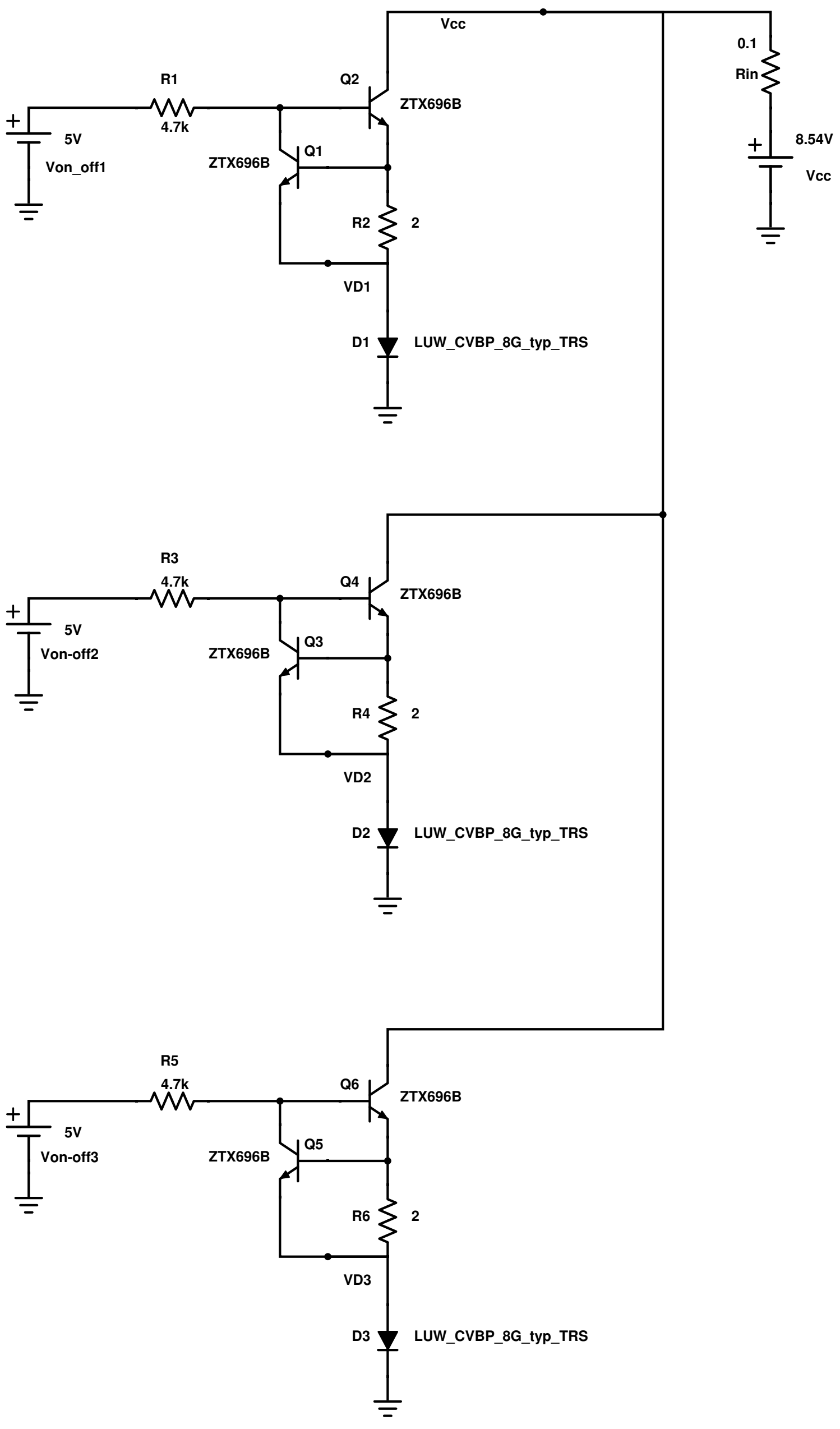

Supplement: S1 File — An archive with all the source files is attached. Please recall that they are available under GPLv2 or later. (ZIP) [file pone.0187219.s005.zip › S1-File/03-LED-drivers/LED-driver.pdf]
